# Associations between follow-up screening after gestational diabetes and early detection of diabetes – a register based study

**DOI:** 10.1186/1471-2458-14-841

**Published:** 2014-08-13

**Authors:** Christinna Rebecca Olesen, Jane Hyldgaard Nielsen, Rikke Nørmark Mortensen, Henrik Bøggild, Christian Torp-Pedersen, Charlotte Overgaard

**Affiliations:** Public Health and Epidemiology Group, Department of Health Science and Technology, Aalborg University, Aalborg, Denmark

**Keywords:** Follow-up screening, Gestational diabetes mellitus, Diabetes mellitus, Risk, Early detection

## Abstract

**Background:**

Women whose pregnancy was complicated by gestational diabetes have a 7-fold higher risk of developing diabetes, primarily type 2. Early detection can prevent or delay the onset of late complications, for which follow-up screening is important. This study investigated the extent of participation in follow-up screening and the possible consequences of nonattendance in the Region of North Jutland, Denmark.

**Method:**

In Danish national registers covering the years 1994–2011 we identified 2171 birthing women whose pregnancy was complicated by first-time gestational diabetes. Control visits to general practitioners and biochemical departments after giving birth were charted. Following national guidelines we defined four intervals for assessment of participation in follow-up screening. Diagnosis of diabetes or treatment with glucose-lowering agents after giving birth were also identified. Participation in follow-up screening and risk of diabetes was calculated. Time to obtaining diagnosis of diabetes or initiating treatment was analysed by Cox regression models. All models were adjusted for age, ethnicity and income.

**Results:**

High attendance was found during the first control interval, after which attendance decreased with time after giving birth for both controls at general practitioners and biochemical departments. All differences in proportions were statistically significant. Women attending controls at general practitioners had a significantly higher risk of diabetes diagnosis and treatment after gestational diabetes than women not attending. The results for women attending testing at biochemical departments also showed an increased risk of initiation of treatment. Women attending at least one general practitioners control had a significantly higher risk of early diabetes diagnosis or treatment. Time to initiation of treatment was significantly higher for testing at biochemical departments. Women with high incomes had a significantly lower risk of diabetes diagnosis or initiation of treatment compared to low-income women.

**Conclusion:**

Participation in follow-up screening after gestational diabetes is low in the North Denmark Region. Follow-up screening ensures early detection of diabetes and initiation of treatment. Our results emphasize the importance of development of interventions to improve early detection and prevention of diabetes after gestational diabetes.

## Background

Women affected by gestational diabetes have a 7-fold higher risk of diabetes compared to women with normoglycaemic pregnancies, with the most rapid increase in type 2 diabetes found during the first five years after giving birth [1, 2]. Diabetes can have serious health implications [3], such as retinopathy, neuropathy and cardiovascular heart disease [4]. Early detection of type 2 diabetes can prevent or delay the onset of late complications [1, 3]. International as well as national guidelines recommend follow-up screening with either oral glucose tolerance tests or fasting plasma glucose tests after pregnancy complicated by gestational diabetes [5, 6]. The participation levels for follow-up screenings reported in the literature ranging from 14 to 61%, are unsatisfying [7]. Various reasons for nonattendance have been found, such as clinicians’ unawareness of the screening offer and women choosing not to participate [8–10]. Cross-national differences in the organization of healthcare systems complicate the applicability of international strategies to increase follow-up screening after gestational diabetes to other healthcare contexts [11]. Public health is compromised by the existence of the pathway from gestational diabetes to type 2 diabetes, which accounts for considerable costs in the healthcare sector [4]. Gestational diabetes may be one of the factors underlying the increasing prevalence of type 2 diabetes worldwide, of which it is considered a valid indicator [3]. Even though the incidence of gestational diabetes is low in Denmark and the other Scandinavian countries, affecting 2–3% of all pregnancies, the preventive potentials of follow-up screening for type 2 diabetes are considerable [1]. A better understanding of the association between screening after gestational diabetes and the subsequent risk of type 2 diabetes is necessary to determine the importance of the screening. Studies regarding participation in follow-up screening in a European context such as Denmark are lacking, which emphasises the importance of this study. The aim of this study was to examine the extent of participation in follow-up screening in The North Denmark Region, and the possible consequences of nonattendance.

## Methods

### Design and setting

A register-based study of gestational diabetes among inhabitants in The North Denmark Region, from 1 January 1994 to 31 December 2011. The administrative Region encompasses around 580.886 inhabitants [12], and as in the rest of Denmark, the health care system is centered around general practitioners [13]. The access to the health care system is free of charge [13]. Denmark’s permanent and unique civil registration number enabled the linkage of individual data across multiple nation-wide registers, known to encompass virtually all inhabitants [14].

### Data sources

The data registers were linked in Statistics Denmark based on the civil registration number. The National Patient Register holds data on all hospital admissions in Denmark [15]. The classification system contains codes that allows the tracing of data associated to specific health care services or diagnoses (International Classification of Diseases, ICD-10), which enabled us to obtain the data relevant for our purposes [16]. The study population was included by the coincidence of birth (ICD-10 codes: D080-D084) [16] and first-time gestational diabetes diagnosis (ICD-10 code: D0244) [16] and/or other diabetes diagnosis (ICD-10 codes: DE10-14) [16] before, during and after pregnancy and birth. Data regarding population characteristics were obtained from the same register. General practitioners are not obliged to register diagnoses in their clinics. Income data were obtained from the Danish Income Statistics Register [14]. The Danish National Prescriptions Registry contains data on all claimed prescriptions from Danish pharmacies [14], and might thus serve as a proxy measure of diabetes. The identification of all claimed prescriptions on glucose-lowering agents (ATC-code: MA10) was enabled by WHO’s ATC classification (Anatomical therapeutic chemical classifications system) [17]. Data from the National Health Service Register [13] enabled the monitoring of the follow-up screening of women whose pregnancy was complicated by first-time gestational diabetes, as general practitioners can perform and analyse oral glucose tolerance tests [18], which are paid by the Health Service. The code for general practitioners testing of blood glucose and/or oral glucose tolerance is the same and was used to identify women who had received follow-up screening (Code: 7136) [18, 19]. Data on all blood samples examined at biochemical departments in the region were obtained to identify women with first-time gestational diabetes who had an oral glucose tolerance test or a fasting plasma glucose test performed since 2006 (Codes: NPU21530; NPU02195) respectively [20]. Both tests are used in the follow-up screening of women with prior gestational diabetes [5]. The NPU terminology (Nomenclature for Properties and Units terminology) is an international coding system that enables identification and communication of the results from clinical laboratories in the health sector [21].

### Data and measurement

For verification of the gestational diabetes (ICD-10 code: DO244) diagnosis, all collected data regarding other diabetes diagnosis (ICD-10 codes: DE10-14) and claimed prescriptions on glucose-lowering agents (ATC-code: MA10) were divided into three subgroups: prior to pregnancy, during pregnancy and after pregnancy. We thus excluded women who had been diagnosed with any type of diabetes and/or treated with glucose-lowering agents prior to pregnancy. Furthermore, exclusion was performed due to the suspicion of underlying unregistered diabetes prior to pregnancy. Claimed prescriptions or a diagnosis within a year after giving birth is known to be related to gestational diabetes and did not lead to exclusion. This approach has been validated in the literature [22]. Women with gestational diabetes that required diet and/or medical treatment were included in the study, despite of their different risk of diabetes after gestational diabetes [6]. International and national guidelines recommend that all women with gestational diabetes-complicated pregnancies participates in follow-up screening 6–8 weeks after giving birth and thereafter at least biannually [5, 6]. Time to control and testing by general practitioners and at biochemical departments were divided into four intervals, defined in accordance with national guidelines [5]. The time ranges for the intervals of follow-up screening at general practitioners and biochemical departments were two years; however, for the last control at biochemical departments a range of approximately four years was constructed. The interval for the first control at general practitioner was 0–3 months after giving birth, for the first control at biochemical departments 0–5 months after giving birth. From this point on the controls will be described as Control 1,2,3,4 for participation at both general practitioners and biochemical departments. Inclusion in the four controls was not conditioned by attendance in any of the other follow-up screening controls. Broad intervals for biochemical department testing were constructed to ensure detection of all women referred from their general practitioner to testing at biochemical departments. The referrals were necessitated by the fact that not all general practitioners perform oral glucose tolerance tests at their clinics. From one year after giving birth we investigated associations between participation in follow-up screening and diabetes diagnosis/treatment with glucose-lowering agents. A time variable was created to analyse associations between attending at least one control and time to diabetes diagnosis or treatment. The variable was based on time from giving birth to obtaining a diagnosis of diabetes or initiation of treatment, until the time limit of the register available or censuring due to next pregnancy/birth or death, whichever occurred first. Data on age and income were divided into tertiles and included as categorical variables. Income was included as a possible confounder since low income is associated with a higher prevalence of diabetes [23]. Income was consumer-indexed (2009), and determined based on data from the year before birth, to ensure the best possible representation of the women’s socioeconomic status. Age was also included as a possible confounder since older women have a higher risk of development of diabetes after gestational diabetes [6]. To allow for differences in the risk of developing type 2 diabetes, three ethnicity categories were created: Caucasian (Danish/other), Asian/Middle Eastern, and African [6, 24]. The categories were constructed because women with Asian, Middle Eastern and African ethnicity has different risks of developing both gestational diabetes and diabetes than Caucasian women [6, 24]. The three variables were included to control for possible confounding regarding diabetes diagnosis or treatment.

### Outcomes

The primary outcome was attendance in follow-up screening at general practitioners or biochemical departments after gestational diabetes in each of the defined time intervals. The outcome was defined by coding of an oral glucose tolerance test or a fasting plasma glucose test. The secondary outcome was the risk of and time to diabetes diagnosis or treatment with glucose-lowering agents among participants and non-participants. Outcomes were predefined.

### Statistical analyses

The study applied descriptive statistics (N/%) to illustrate the distribution of age, income and ethnicity. All models were adjusted for these variables. Participation in follow-up screening was analysed using chi-squared tests. Women appearing as missing in the tables were either excluded or censured by death, second birth or end of registration. To determine the association between participation in follow-up screening and diabetes-related outcomes, we applied logistic regression models presented with odds ratios (OR) and 95% confidence intervals (CI). We furthermore examined possible confounding by age, income and ethnicity and interactions between ethnicity and income, and between age and income. Cox’s proportional hazard models were applied to investigate the hazard ratio (HR) of diabetes related outcomes, depending on participation in at least one general practitioner or biochemical department control. All Cox proportional hazard models met the proportional hazard assumption. The SAS statistical software package for Windows, version 9.2, was used (SAS Institute, Cary, NC, USA).

### Ethics

Register based observational studies do not require ethical approval in Denmark [25]. The Danish Data Protection Agency approved this study (No. 2007-41-1667).

## Results

### Participant characteristics

**Figure 1:**
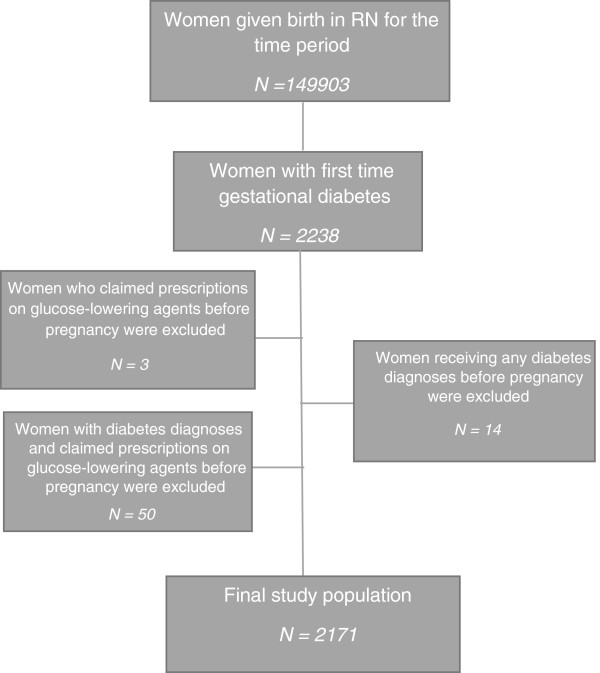
**Flowchart.**

A total of 149.903 women in The North Denmark Region gave birth during the study period, 2238 of whom had a diagnose of first-time gestational diabetes. Diagnosis of diabetes and/or treatment with glucose-lowering agents prior to pregnancy lead to the exclusion of 67 women. The final study population included 2171 women (Figure 1). As shown in Table 1 the study population had a mean age of 30.9 years and the mean number of births at the time of first gestational diabetes diagnosis was 1.6. Baseline characteristics on income, ethnicity and age are also listed in Table 1. Among the women in the Region 1.4% had first-time gestational diabetes during the time period.

**Table 1:** **Population characteristics**

Variables	N (%)
**Women**	2171 (100)
Age*	
Young	717 (33)
Middle	716 (33)
Older	738 (34)
Income**	
Low	715 (33)
Middle	714 (33)
High	715 (34)
**Ethnicity**	
Caucasian (Danish/other)	1981 (92.3)
Asian/middle eastern	128 (5.9)
African	61 (2.8)
**Mean**	**SD**
Birth of diagnosis	0.8
1.6	
Age	5.0
30.9	

*Age categorized as tertiles: Young: <=29.7 Middle: >29.7.

<=33.9 and older > 33.9.

**Income categorized as tertiles (Dk.kr.) Low: <=292.872.

Middle: >292.872 and < =395.691 High: >395.691.

### Participation in follow-up screening

Table 2 shows the participation in follow-up screening. Participation decreased with time after giving birth. The proportion of women attending Control 1 at their general practitioner after giving birth was 80.5%, 47.3% attended Control 2, 29.1% Control 3 while 17.7% attended Control 4. For controls conducted at biochemical departments, the proportion of women attending decreased from 10.2% at Control 1 to 0.5% at Control 4. Significant differences were found for all proportions regarding attendance and non-attendance, with p-values under the 5% significance level (Table 2). The number of women categorized as missing increased primarily due to the occurrence of a subsequent pregnancy. Of the 2171 women in the study, 88 (4.1%) had treatment-requiring gestational diabetes (not shown in tables). One-hundred and twenty-four (5.8%) were subsequently diagnosed with diabetes and 229 (11%) claimed prescriptions on glucose-lowering agents after giving birth (Table 2). To estimate undiagnosed diabetes among non-participants, we applied the mean number of diabetes diagnosis among the participants; yielding an estimated 119 non-attenders to general practitioner controls and 509 non-attenders to biochemical departments with undiagnosed diabetes (not shown in tables).

**Table 2:** **Follow-up screening participation after gestational diabetes in the Region in the period 1994-2011**

Follow-up screening					Diabetes women (N = 124)			Treatment women (N = 229)		
	Control + N (%)	Control – N (%)	Missing N (%)*	p	Control + N	Control – N	p	Control + N	Control – N	p
**General practitioner (N = 2171)**										
Control 1: Within 3 months after birth	1744 (80.5)	423 (19.5)	41 (0.2)	<0.001	112	12	0.0045	205	24	0.0002
Control 2: 3 months to 2 years	852 (47.3)	950 (52.7)	369 (17.0)	0.0210	108	16	<0.0001	188	37	<0.0001
Control 3: 2 years to 4 years	526 (29.1)	1281 (70.9)	364 (16.8)	<0.001	94	27	<0.0001	152	62	<0.0001
Control 4: 4 years to 6 years	333 (17.7)	1551 (82.3)	287 (12.8)	<0.001	75	37	<0.0001	116	89	<0.0001
Biochemical unit** (N = 1218)										
Control 1: Within 6 months after birth	111 (10.2)	979 (89.8)	128 (10.5)	<0.001	113	10	0.4932	181	40	<0.0001
Control 2: 6 moths to 2.4 years	69 (6.4)	1013 (93.6)	136 (11.2)	<0.001	113	10	0.8829	184	36	<0.0001
Control 3: 2.4 years to 4.5 years	29 (2.5)	1116 (97.5)	73 (6.0)	<0.001	113	9	0.3085	192	28	<0.0001
Control 4: 4.5 years to 8.2 years	6 (0.5)	1190 (99.5)	22 (1.8)	<0.001	115	5	0.7766	198	22	<0.0001

*Women appearing as missing in the table were censured by either new pregnancy or birth and death.

**Data on blood samples at biochemical departments are only available for women giving birth after 2006.

Logistic regression analysis results showed a statistical significant positive association between attendance at general practitioner controls and receiving a diagnosis of diabetes and initiation of treatment with glucose-lowering agents after gestational diabetes (Tables 3 and 4). The lowest risk applied to Control 1 for diabetes diagnosis (OR 2.4; 95% CI 1.3-4.4) and the highest risk applied to Control 4 for diabetes diagnosis (OR 11.8; 95% CI 7.7-18.0) (Tables 3). Associations between diagnosis of diabetes and follow-up screening at biochemical departments were insignificant for all controls (Table 3).

**Table 3:** **Association between participation in follow-up screening and diabetes diagnosis**

	Crude OR (95% CI)	Adjusted* OR (95% CI)
**General practitioner**		
Control 1: Within 3 months after birth	2.4(1.3-4.3)	2.4 (1.3-4.4)
Control 2: 3 months to 2 years	8.6 (5.1-14.7)	8.3 (4.8-14.1)
Control 3: 2 years to 4 years	10.1 (6.5-15.7)	9.8 (6.2-15.3)
Control 4: 4 years to 6 years	11.9 (7.9-18.0)	11.8 (7.7-18.0)
Biochemical unit**		
Control 1: Within 6 months after birth	1.4 (0.4-4.9)	0.9 (0.2-4.1)
Control 2: 6 months to 2.4 years	2.4 (0.7-8.2)	1.5 (0.3-6.9)
Control 3: 2.4 years to 4.5 years	4.3 (0.9-19.2)	1.7 (0.2-14)
Control 4: 4.5 years to 8.2 years***	-	-

Women with onset of new pregnancy/birth or occurrence of death were excluded from the analysis.

*Adjusted for income, age and ethnicity.

**Data on blood samples are only available for women giving birth after 2006.

***To few events.

**Table 4:** **Associations between participation in follow-up screening and treatment with glucose lowering agents**

	Crude OR (95% CI)	Adjusted* OR (95% CI)
**General practitioner**		
Control 1: Within 3 months after birth	2.2 (1.5-3.5)	2.3 (1.5-3.5)
Control 2: 3 months to 2 years	7.4 (5.1-10.6)	7.1 (4.9-10.3)
Control 3: 2 years to 4 years	8.1 (5.9-11.2)	8.1 (5.9-11.3)
Control 4: 4 years to 6 years	8.8 (6.5-12.1)	8.9 (6.5-12.2)
Biochemical unit**		
Control 1: Within 6 months after birth	3.1 (1.7-5.5)	2.9 (1.6-5.3)
Control 2: 6 months to 2.4 years	3.8 (2.0-7.3)	3.5 (1.8-6.9)
Control 3: 2.4 years to 4.5 years	3.3 (1.2-8.9)	2.5 (0.8-7.4)
Control 4: 4.5 years to 8.2 years	7.9 (1.4-43.8)	8.7 (1.5-49.4)

Women with onset of new pregnancy/birth or occurrence of death were excluded from the analysis.

*Adjusted for income, age and ethnicity.

**Data on blood samples are only available for women giving birth after 2006.

The lowest risk also applied for Control 1 for treatment outcomes (OR 2.3; 95% CI 1.5-3.5) the highest risk applied for Control 4 for treatment (OR 8.9; CI 95% 6.5-12.2) (Tables 4). A significant positive association between testing at a biochemical department and treatment with glucose-lowering agent was also detected, with the lowest values applying for Control 1 (OR 2.9; 95% CI 1.6-5.3) and the highest for Control 4 (OR 8.7; 95% CI 1.5-49.4). However, the association was insignificant for Control 3 (Table 4). Confidence intervals (Tables 3 and 4) widely increased with time, due to the decreasing attendance at either type of testing place.

**Figure 2:**
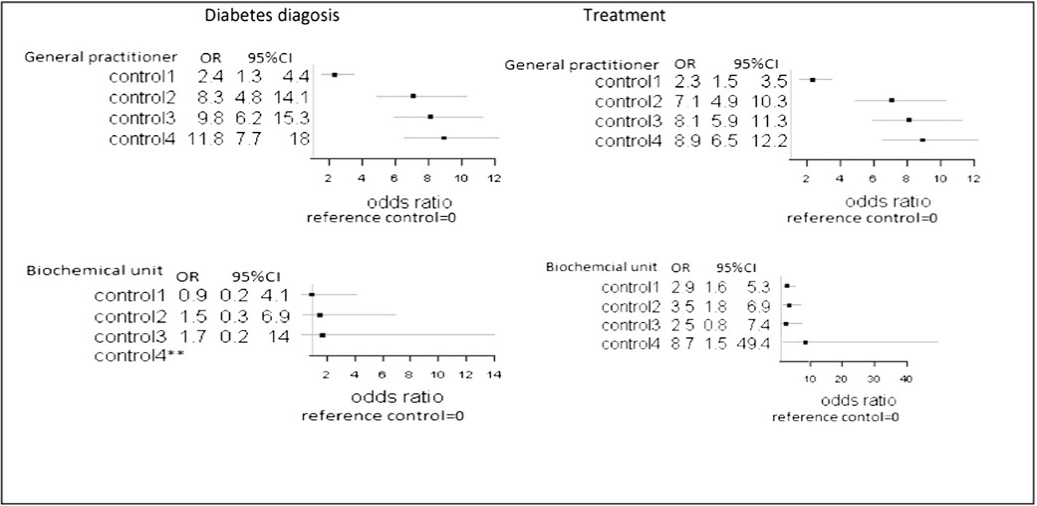
Risk of diabetes diagnosis or treatment for control at general practitioner or biochemical department*. *Adjusted for income, age and ethnicity. Data on blood samples are only available for women giving birth after 2006. **To few events. Women with onset of new pregnancy/birth or occurrence of death were excluded from the analysis.

The forest plots in Figure 2 illustrates the risk of diabetes diagnosis or initiation of treatment. Income was a confounding factor, with a negative association between income and diagnosis of diabetes (not shown in tables). Women with the highest incomes had a statistically significant lower risk of being diagnosed with diabetes (OR 0.6; 95% CI 0.4-0.9) or of being treated with glucose-lowering agents (OR 0.6; 95% CI 0.5-0.9) compared to women with the lowest income. No interaction between ethnicity and income, or between ethnicity and age, was detected.

Results of the Cox regression analysis showed statistically significant positive associations between attendance to at least one control at either a general practitioner or a biochemical department and time to receiving a diabetes diagnosis or initiation of treatment (Table 5). Women attending at least one general practitioner control had a higher risk of diabetes diagnosis (HR 2.7; 95% CI 1.1-5.9) or treatment with glucose-lowering agents (HR 2.1; 95% CI 1.2-3.5) compared to women who did not attend either of the controls. The risk of treatment with glucose-lowering agents was also higher for women attending only one control at a biochemical department (HR 2.1; 95% CI 1.2-3.6). Values for the risk of diabetes diagnosis for the women attending a biochemical department did not reach the significance level (Table 5). High-income women had a significantly shorter time to diabetes diagnosis (HR 0.6; 95% CI 0.4-0.9) or treatment (HR 0.7; 95% CI 0.5-0.9) compared to women with the lowest incomes (not shown in tables). The association between time to diabetes or treatment was not influenced by interaction.

**Table 5:** **Association between risk-time to diabetes diagnosis or treatment at minimum one control**

		Diabetes			Treatment	
	N	Crude	Adjusted	N	Crude	Adjusted
		HR (95% CI)	HR (95% CI)*		HR (95% CI)	HR (95% CI)
Min. 1 control at general practitioner (n = 2123)	124/2123	2.6 (1.15-5.9)	2.7 (1.1-5.9)	229/2089	2.1 (1.2-3.5)	2.1 (1.2-3.5)
Min. 1 control at a biochemical unit**	22/1211	1.1 (0.3-3.9)	0.8 (0.1-3.3)	77/1202	2.2 (1.3-3.8)	2.1 (1.2-3.6)

Women with onset of new pregnancy/birth or occurrence of death were excluded from the analysis.

*Adjusted for income, age and ethnicity.

**Data on blood samples are only available for women giving birth after 2006.

## Discussion

This study shows low participation in follow-up screening after gestational diabetes that are recommended in international and national guidelines [5, 6]. The study of 2171 women with first-time gestational diabetes showed significantly decreasing participation in follow-up screening, from 80.5% at Control 1 to 17.7% of the women visiting their general practitioner for Control 4. Even lower attendance was found for biochemical departments, with 10.2% at Control 1 and 0.5% attending Control 4. The risk of a diabetes diagnosis was strongly associated with participation in follow-up screening, with an 11.8-fold increased risk for women attending general practitioner Control 4. Attenders at Control 4 had an increased risk of initiation of treatment with glucose-lowering agents at 8.9-fold and 8.7-fold for general practitioner and biochemical department controls, respectively. Women who attended at least one general practitioner control moreover had a 2.7-fold increased risk of shorter time to diabetes diagnosis. This also applied for the initiation of treatment, with a 2.1-fold increase in the risk for either type of control. The high risk of diabetes diagnosis and initiation of treatment among women attending the controls emphasizes that attendance enables early detection of diabetes, which can prevent the development of late complications [1, 3].

### Strengths and limitations

The strength of this study is its basis in the complete and comprehensive data held by the Danish national registers and the negligible risk of loss to follow-up [14, 26]. The structure of the Danish health care system and the possibility to include both hospital and general practitioners, as well as both register based diagnosis and initiated treatment allows a full follow-up on a large cohort. The defined intervals were furthermore sufficiently broad to ensure the detection of women who participated in follow-up screenings, whether at their general practitioner or at biochemical departments. The limitations of the study relates to the validity problems in connection with the regionally based blood sample registration, since this serves purely administrative purposes. We are unaware of any validity studies of on the regional blood sample registration. Our results on the proportion of women having tests performed at Control 1 are considerably higher than those found in comparable studies [7, 8, 11, 27]. However, data on blood sampling have only been available since 2006, which has limited the analysis of testing at biochemical departments to women giving birth after that time. The insignificant results obtained for diagnosis of diabetes as outcome may be caused by the limited data. The problem of under-registration of diabetes diagnosis was caused by the fact that not all women in treatment with glucose-lowering agents had a registered diabetes diagnosis. Inaccuracies in coding procedures may have led to the problematic validity of diabetes diagnosis registered in the National Patient Register [28]. However, the National Patient Register enabled detailed information regarding hospital admissions in Denmark and further description of the register can be obtained from Lynge et. al. [28]. The detailed information of the National Prescription Registry, however, enabled us to detect women in diabetes treatment who were not registered with a diabetes diagnosis. Further information regarding the National Prescription registry can be obtained from Kildemoes et. al. [26]. As described earlier, type 2 diabetes is the more frequent diabetes type among women with previous gestational diabetes [26]. We expected that the majority of the diagnosed women had received a type 2 diabetes diagnosis. The diabetes outcome category contains all diagnosis of diabetes, which strengthens the validity of our study and prevented us from excluding women diagnosed with diabetes after gestational diabetes. The use of the National Health Service Register may also have caused validity problems, although we are unaware of any relevant studies regarding this matter. Further description regarding this register can be obtained from Andersen et. al. [13]. Despite the strong economic incentives, the misreporting of minor additional laboratory services in particular, may occur since fees for these services are small [13]. Whether the general practitioner performed oral glucose tolerance tests or blood glucose tests cannot be ascertained, as the codes used to detect follow-up screening are the same [18, 19]. General practitioners who are unable to perform or analyse oral glucose tolerance tests can refer the women or have the test performed by a biochemical department. However, 80.5% of the women were registered as having a test performed at Control 1, which indicates a predominantly correct coding. A further limitation of this study concerns the possible demographic selection bias related to its basis in the relatively deprived North Denmark Region [12, 29]. A higher proportion of socioeconomically disadvantaged women in our population may have led to an overestimated risk of diabetes related outcomes in relation to non-attendance in follow-up screening, since socioeconomic status is a determinant for diabetes [23]. Results regarding the risk of diabetes can be higher in our study population, and the relatively high risks have to be interpreted with caution. However a previous mentioned meta-analysis finds the risk of diabetes among women with pregnancy complicated by gestational diabetes, 7-fold higher compared to women with normoglycaemic pregnancies [1]. Only at the later controls our risk estimates exceeds the risk found in the meta-analysis.

### Interpretation

While we found high participation rates in the first control after gestational diabetes, other studies have reported rates varying from 14 to 61% for this control. A recent review also finds that screening for diabetes after gestational diabetes generally is low [30]. However, those studies were restricted to the first control [11]. Organisational differences between health care sectors effects the comparability with results from other health care contexts. Attendance varied greatly for testing at first control, with 80.5% and 10.2% for general practitioners and biochemical departments, respectively. The majority of results retained significant levels, however the limited data on blood samples may have caused insignificant associations between controls at biochemical departments and diagnosis of diabetes and treatment at Control 3. A likely reason for the high participation in the first general practitioner control after giving birth is that this examination is part of a standard postpartum visit [31]. General practitioner controls had higher attendance than biochemical department controls, possibly because some practitioners are capable of performing the oral glucose tolerance test. However, other regions recommend that the tests are analysed or performed only by biochemical departments because of the varying quality of the general practitioners equipment [32]. Low participation in follow-up screening can be related to healthcare providers, health systems and patient barriers. Barriers related to healthcare systems concerns different practice between countries. Healthcare providers can be unaware of the guidelines and lack of communication between healthcare providers. General practitioners can also fail to perform the test or refer the women to biochemical departments [8–10, 27]. The patient related barriers are lack of time and concerns about future health [30]. Women can also be unaware of the risk of diabetes after gestational diabetes [8–10, 27]. The fragmentation of care may also account for low participation since this often leads to confusion about the responsibility for follow-up screening [8–10, 27, 33]. Participation gives women a higher risk of diabetes diagnosis or treatment and an increased possibility of early detection of diabetes or initiation of treatment. The risk estimates were high at the last controls possibly due to the attendance of women who had been diagnosed or treated at earlier controls, although their attendance also decreased over time. The women who were not followed up were more likely to be diagnosed at a later point, when their diabetes may have progressed, since clinical diabetes can remain undiagnosed for years [22]. The high risk of a diabetes diagnosis among women attending controls and estimated undiagnosed diabetes among non-attending women, lead us to believe that a considerable number of the non-attenders can be affected by diabetes after gestational diabetes. We found the diabetes risk to increase with successive controls, which is in accordance with findings from other studies that show an increasing risk of diabetes within the first five years after gestational diabetes [2]. Women with high income had a significantly lower risk of diabetes diagnosis or treatment. Socioeconomic factors are plausible reasons for this, since they are one of the determinants for diabetes [23]. In view of the limitations described in previous sections, our results should be interpreted with caution. The main result of this study is the finding of low participation in follow-up screening after gestational diabetes, except for the first control after giving birth. Our study has demonstrated the importance of follow-up screening of women to ensure early detection and initiation of treatment for the prevention of late complications caused by diabetes. Follow-up screening of these women is a concern for public health as low participation impedes the detection of diabetes in women with previous gestational diabetes. This has been emphasized by a previous Danish study, which found that two out of three diabetes cases in the general population were undiagnosed [34]. The low participation in follow-up screening after gestational diabetes may reflect an ignorance of the risk involved, or failure by health staff to refer the patient and unawareness of guidelines [8–10]. Fragmented care is another possible factor since the care of women is organised between obstetricians and general practitioners. The fragmentation can contribute to lack of communication regarding the recommendations for follow-up screening and the women’s future risk between the providers [8–10, 33]. The study provides clinicians with important knowledge of the risk of diabetes among non-attending women and the need for communicating the risk to the women in order to ensure follow-up screening. In low-income countries, women with pregnancy complicated by gestational diabetes face greater barriers such as availability, affordability and access to services, which concerns both screening during pregnancy and access to follow-up screening [30].

### What this study adds

In contrast to other studies examining participation in follow-up screening [7, 8, 11, 27, 35], this study followed women with previous gestational diabetes over several screening rounds and examined the consequences of non-attendance in follow-up screening. We document rapidly declining participation in follow-up screening with time and estimated that women not attending potentially can have undiagnosed diabetes. This study also shows that follow-up screening enables early detection of diabetes. Our study furthermore demonstrates the great potential of monitoring the quality of health care sectors through national registers.

### Suggestions for further research

Our study has documented the need for preventive health interventions to ensure stringent follow-up screening of women with prior gestational diabetes in order to ensure the early detection of diabetes and initiation of treatment. The general lack of interventions to ensure participation in follow-up screening is described in other studies [8, 27, 33, 35]. Our findings may furthermore help in the development of interventions to enhance participation in follow-up screening. Possible interventions could concern reduction of fragmented care and facilitation of other efforts to improve participation in follow-up screening. Such efforts could concern reminder systems, which has shown to improve the participation in follow-up screening significantly [8, 11, 35, 36]. Such systems can have a positive impact on the participation in follow-up screening [31].

## Conclusion

In this study of women with prior first-time gestational diabetes we found low participation rates in follow-up screening, except for the first control after which it decreased with time after giving birth. Women who did not attend follow-up screening had a lower risk of diabetes diagnosis or treatment and early detection of diabetes. The higher possibility of early detection of diabetes among women attending follow-up screening reduces their risk of late complications. Contrary to national and international guidelines [5, 6], the majority of women with gestational diabetes failed to return for follow-up screening, which emphasizes the importance of interventions to ensure early detection and prevention of diabetes after gestational diabetes [8, 11, 35].
